# Establishing electroporation thresholds for targeted cell specific cardiac ablation in a 2D culture model

**DOI:** 10.1111/jce.15641

**Published:** 2022-08-16

**Authors:** Sahar Avazzadeh, Mahshid H. Dehkordi, Peter Owens, Amirhossein Jalali, Barry O'Brien, Ken Coffey, Martin O'Halloran, Howard O. Fernhead, David Keane, Leo. R Quinlan

**Affiliations:** ^1^ Physiology and Cellular Physiology Research Laboratory, CÚRAM SFI Centre for Research in Medical Devices, School of Medicine, Human biology building National University of Ireland (NUI) Galway Ireland; ^2^ Department Pharmacology and Therapeutics, School of Medicine, Biomedical Research Building National University of Ireland (NUI) Galway Ireland; ^3^ Centre for Microscopy and Imaging, Human Biology Building National University of Ireland (NUI) Galway Ireland; ^4^ Department of Mathematics and statistics University of limerick Limerick Ireland; ^5^ AtriAN Medical Limited, Unit 204 NUIG Business Innovation Centre Galway Ireland; ^6^ Translational Medical Devise Lab (TMDLab), Lambe Institute of Translational Research University College Hospital Galway Galway Ireland; ^7^ Electrical & Electronic Engineering, School of Engineering National University of Ireland Galway Galway Ireland; ^8^ Cardiac Arrhythmia Service, St Vincent's University Hospital Dublin Ireland

**Keywords:** atrial fibrillation, cardiac ablation, irreversible electroporation

## Abstract

**Background:**

Irreversible electroporation has emerged as a new modality to overcome issues associated with other energy sources for cardiac ablation. Strong evidence on the optimal, effective, and selective voltage threshold is lacking for both in vitro and preclinical in vivo studies. The aim of this study is to examine the optimal threshold for selective cell ablation on cardiac associated cell types.

**Methods:**

Conventional monophasic and biphasic pulses of different field strength were delivered in a monolayer culture system of cardiomyocytes, neurons, and adipocytes. The dynamics of cell death mechanisms were examined at different time points.

**Results:**

Neurons exhibit higher susceptibility to electroporation and cell death at higher field strength of 1250 V/cm in comparison to cardiomyocytes. Cardiac adipocytes showed lower susceptibility to electroporation in comparison to other cell types. A significant proportion of cardiomyocytes recovered after 24 h postelectroporation, while neuronal cell death remained consistent but with a significant delayed cell death at a higher voltage threshold. Caspase 3/7 activity was observed in both cardiomyocytes and neurons, with a higher level of activity in cardiomyocytes in response to electroporation. Biphasic and monophasic pulses showed no significant difference in both cell types, and significantly lower cell death in neurons when inter pulse interval was reduced.

**Conclusions:**

This study presents important findings on the differences in the susceptibility of neurons and cardiomyocytes to irreversible electroporation. Cell type alone yielded selective and different dynamics in terms of the evolution and signaling mechanism of cell death in response to electroporation.

## 1 INTRODUCTION

Atrial fibrillation (AF) is the most prevalent cardiac arrhythmia, affecting approximately 2% of the population, increasing to 9% in those aged over 65.^1^ Isolation of the pulmonary veins (PVI) by percutaneous catheter ablation has become an established treatment for AF. However, energy sources in catheter ablation to date result in collateral damage to adjacent structures such as the oesophagus and phrenic nerve due to uncontrolled negative thermal effects. These concerns have resulted in a generally conservative approach to energy delivery such that efficacy in terms of lesion completeness and durability is often compromised, leading to only a modest success rate for PVI.

Over the last decade, electroporation has been explored as a biophysical intervention where electrical pulses of a nanosecond to millisecond duration have been shown to alter membrane permeability. Irreversible electroporation (IRE) has proved to be a safe and effective technique as a minimally‐thermal ablation approach. The application of IRE for cardiac ablation has been studied in both in vivo preclinical animal and in human studies.^2^, ^3^, ^4^, ^5^ Extensive early preclinical work demonstrated how pulsed electric fields can be delivered with minimal risk of damage to surrounding structures, such as the coronary arteries,^6^ the esophagus^7^ and the phrenic nerve,^8^ while creating extensive myocardial lesions. Separate to developments in using electroporation to achieve PVI, there is parallel interest in the potential benefits of targeted ganglionated plexi (GP) ablation for the treatment of AF.^9^ Significant effort has been made over a number of years in understanding the initiation of the electrical imbalance, and the cellular and molecular mechanism responsible for the electrophysiological changes observed in AF patients.^10^ Alterations in the autonomic nervous system have been recognized as a key element in the initiation and maintenance of AF.^10^, ^11^ With this, comes the possibility of selectively ablating GPs with IRE resulting in minimal myocardial or collateral structure damage.^12^

The goal of the current study is to assess cell selectivity and ablation thresholds in cardiomyocytes, neurons, and adipocytes in a 2D in vitro model system. Furthermore, we investigate the temporal dynamics of IRE‐induced cell death and associated pathways following electroporation.

## 2 MATERIALS AND METHODS

### 2.1 Cell culture

Mouse atrial cardiomyocytes HL‐1 (Sigma‐Aldrich) and PC12 neurons (ATCC, pheochromocytoma cells derived from *Rattus norvegicus* adrenal glands) were cultured in T75 flasks and passaged with trypsin‐ethylenediaminetetraacetic acid (EDTA) 0.025% (Sigma‐Aldrich) every 2–3 days for maintenance. PC12 cells were cultured in DMEM (Sigma‐Aldrich), supplemented with 10% fetal bovine serum (Sigma‐Aldrich) and 1% penicillin/streptomycin (10,000 U/ml, Gibco). For differentiation, cells were seeded at a density of 6 × 10^4^ cells/ml and 2 h later changed to differentiating medium consisting of OptiMEM (31985062; Thermo Fisher Scientific), supplemented with 0.5% fetal bovine serum, 1% penicillin/streptomycin and 50 ng/ml nerve growth factor (A42578; Thermo Fisher Scientific). Differentiation medium was changed every other day over 7 days. Cardiomyocytes were cultured in Claycomb medium, supplemented with 10% fetal bovine serum, 1% penicillin/streptomycin, 2 mM l‐glutamine and 0.1 mM norepinephrine (Sigma‐Aldrich). Human adult subcutaneous preadipocytes (8025‐05A; Sigma‐Aldrich,) were cultured in T75 flasks in human pre‐adipocyte growth medium (811‐500; Sigma‐Aldrich) and subcultured every 4/5 days with trypsin‐EDTA 0.025%. Adipocytes were differentiated at density of 9 × 10^4^ in a 24‐well plate in differentiation medium (811D‐250) over 2 weeks with media changes every 2 days.

### 2.2 Immunocytochemistry and staining

Neurons and cardiomyocytes were fixed with 4% paraformaldehyde for 20 min, blocked with 0.2% bovine serum albumin (in 0.1% trition‐X100) for 1 h at room temperature. Neurons and cardiomyocytes were incubated with TUJ1 (Ab78078; Abcam) and myosin 4 monoclonal antibody (Thermo Fisher Scientific) respectively in blocking solution at 4°C overnight. Primary antibodies were aspirated and incubated with anti‐mouse 488 fluorophore conjugated secondary antibody (SAB4600387, 77671‐1ML‐F, 1:1000; Sigma) and DAPI in blocking solution for 1 h at room temperature. Cells were imaged using an EVOS M7000 microscope system (Thermo Fisher Scientific). Differentiated adipocytes were stained with prefiltered oil red O in 6:4 ratio in deionized water for 10 min at room temperature. Cells were then fixed in 4% paraformaldehyde for 15 min, washed in distilled water and rinsed with 60% isopropanol, before washing in water three times and imaged in brightfield.

### 2.3 Electroporation protocols

**Figure 1:**
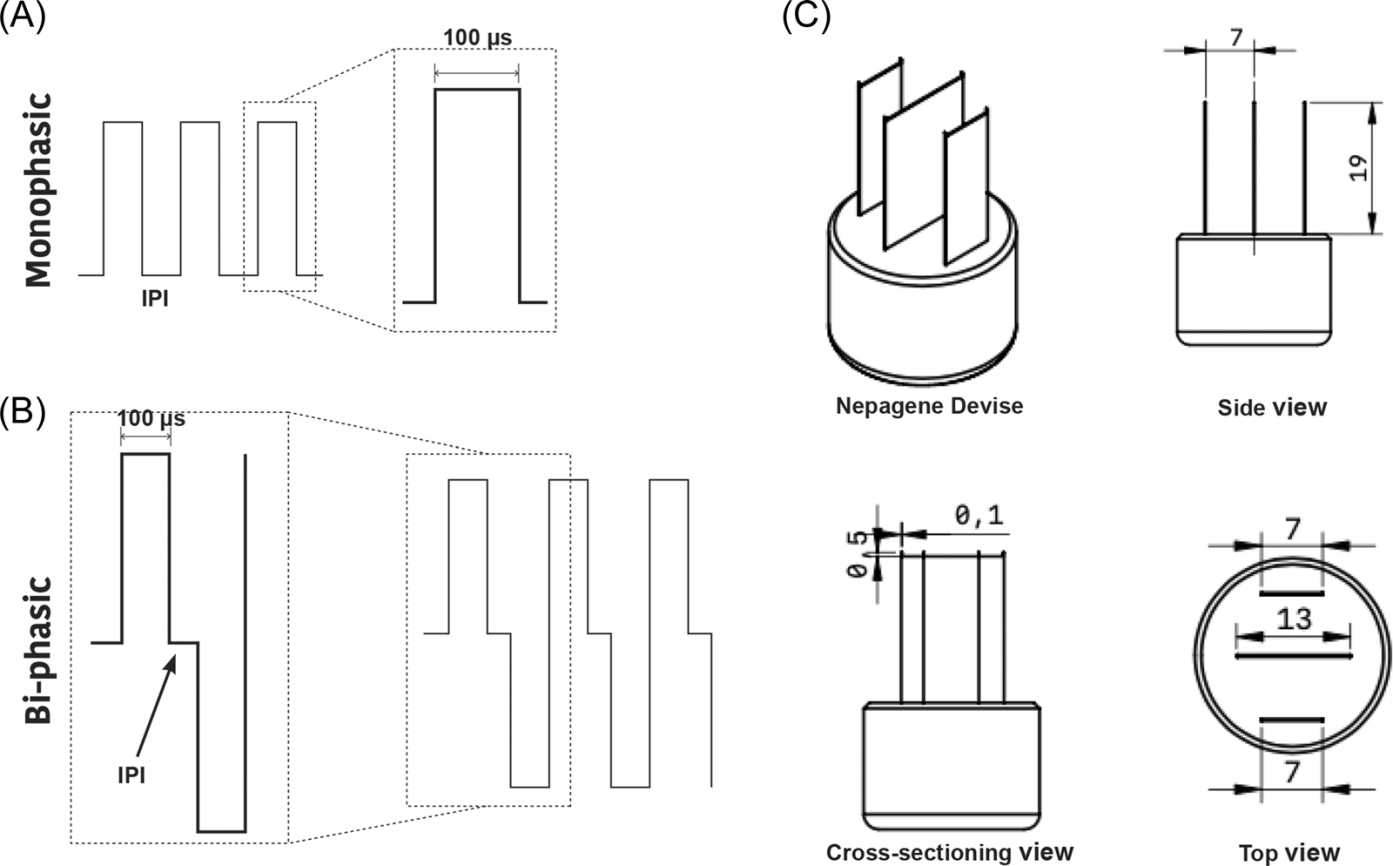
Electroporation waveforms and electrode setup. (A) Monophasic pulses (30, 60) with an inter‐pulse interval (IPI) of 1 s and 1000 or 1250 V/cm or (B) biphasic pulses (30, 60) of 100 µs duration and 0.05 s IPI and 1000 or 1250 V/cm (for each phase) applied to 2D adherent cells. (C) Cell adherent electroporation device (Nepagane) was used to create electric between across a 5 mm gap

Depending on the cell type, previously optimized cell densities were used. Differentiated PC12‐neurons at initial density of 6 × 10^4^, cardiomyocytes at 15 × 10^4^ and differentiated adipocytes at initial cell density of 9 × 10^4^ cells/ml all cases cultures were exposed to a typical IRE protocol of 100 μs duration, monophasic (Figure 1A) or biphasic pulses (Figure 1B) with different inter‐pulse intervals (1, 0.5, 0.05 s). The electric fields were generated using an electroporation device designed for adherent cell cultures (Nepa Gene Co.) (Figure 1C). The electrode was typically placed in the single well of 24‐well plate with a separation of 5 mm between the electrode centers. Field strength was either 1000 or 1250 V/cm applied in pulses of numbers of 30 or 60 per burst.

### 2.4 Live‐dead assay

Before electroporation, culture media was replaced with 400 µl of fresh medium. After electroporation, cells were incubated with 1.5 µM propidium iodide (PI, 30 min at 37°C) (Sigma‐Aldrich) at the desired timepoint (0.5, 3, or 24 h). PI is a red‐florescent nuclear counterstain which is commonly used to detect death cells by binding to their DNA. The PI signal was measured using Hidex microplate reader (Hidex Sense) at an excitation/emission of 520/620 nm. PI positive (PI^+^) cells were also examined in full field of view images using the Operetta high content imaging system (PerkinElmer. Inc) at ×20. The percentage of PI^+^ cells was normalized against DAPI (nuclear staining) and quantified using commercial software (harmony® high content imaging). The lesion area was determined as the area of PI^+^ cells using ImageJ.

### 2.5 Live cell imaging caspase 3/7 assay

Neurons and cardiomyocytes were cultured in 24‐well plates and after electroporation (1000 V/cm or 1250 V/cm, 60 pulses) incubated further with 2.5 µM Incucyte caspase 3/7 (4704; Starius) for 30 min at 37°C. Caspase 3/7 are activated during apoptosis cell death mechanism and this green, fluorescent activity can be monitored by binding of caspase 3/7 dye to the nuclear DNA of apoptotic cells. Caspase 3/7 activity was imaged using the FV3000 Olympus confocal microscope in an imaging chamber at 37°C and 5% CO_2_ over 10 h. Images were captured every hour for 16 fields of view across each well. The percentage of caspase positive cells were analyzed with CellSens (Version 3.2). An adaptive threshold was applied for segmentation with threshold of the average size of pycnotic apoptotic cells at 30 µm^2^. The average total number cell number was calculated from control fields of view using the cell counter plugin in ImageJ.

### 2.6 Statistical analysis

All data were analyzed using two‐way analysis of variance (ANOVA) or repeated measure two‐way ANOVA (Tukey multiple comparison test) using GraphPad. Caspase activity analysis over time were performed in R package (version 4.0.3) using repeated measure ANOVA method to investigate the main effects and the interaction effects of cell type, time, and treatment. In addition, post‐hoc comparison (Tukey) was performed to identify the level of significance observed in repeated measure ANOVA. The Bonferroni correction was also applied to prevent the inflation of test error, in which the *p* values are multiplied by the number of comparisons. All experiments were repeated for at least three independent experimental blocks.

## 3 RESULTS

### 3.1 Effect of time on cell death after electroporation

**Figure 2:**
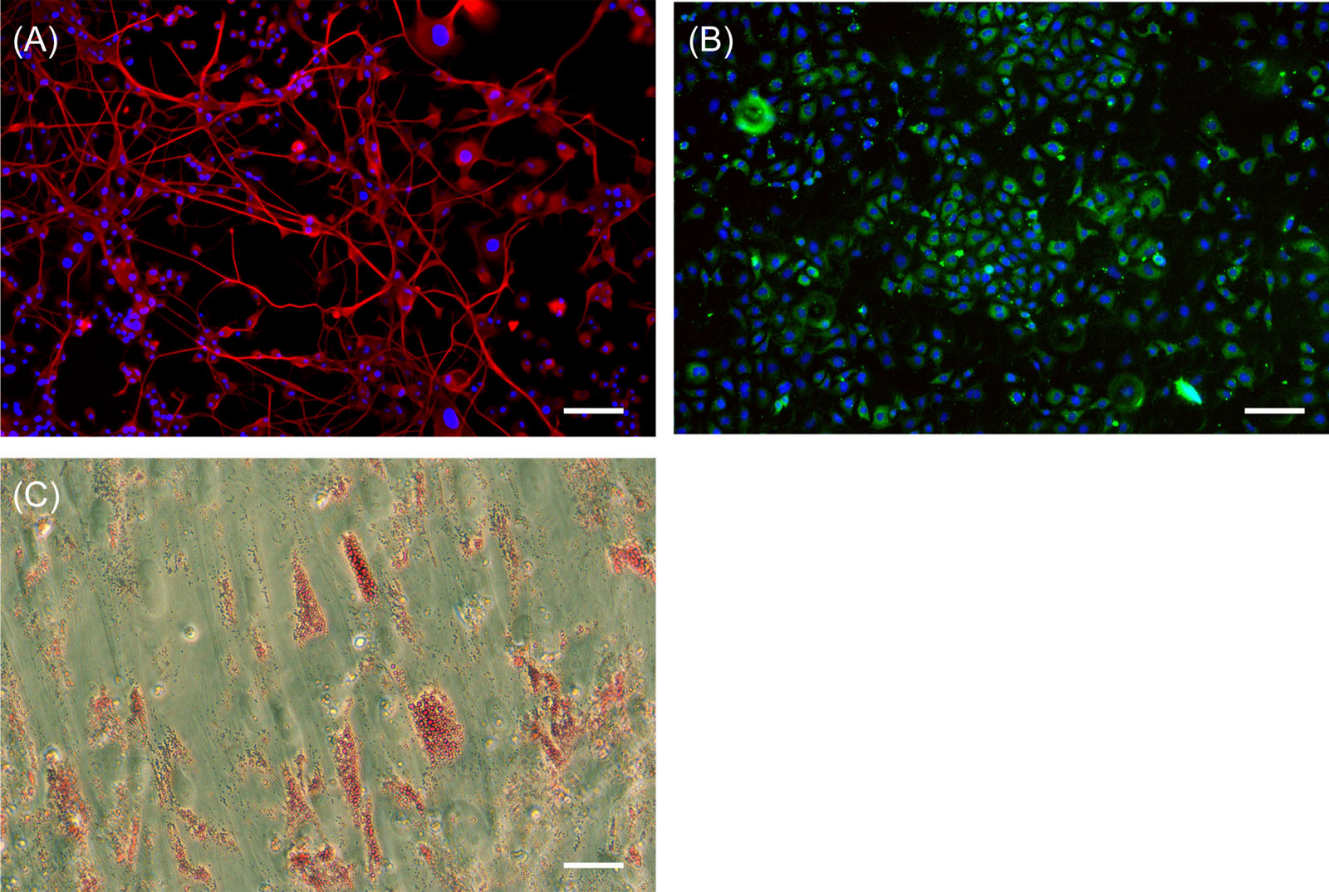
Characterization of cell lines used in the study. (A) differentiated PC12‐neurons (B) HL‐1 mouse atrial cardiomyocytes and (C) differentiated human adipocyte. Scale bar at 100 µM

**Figure 3:**
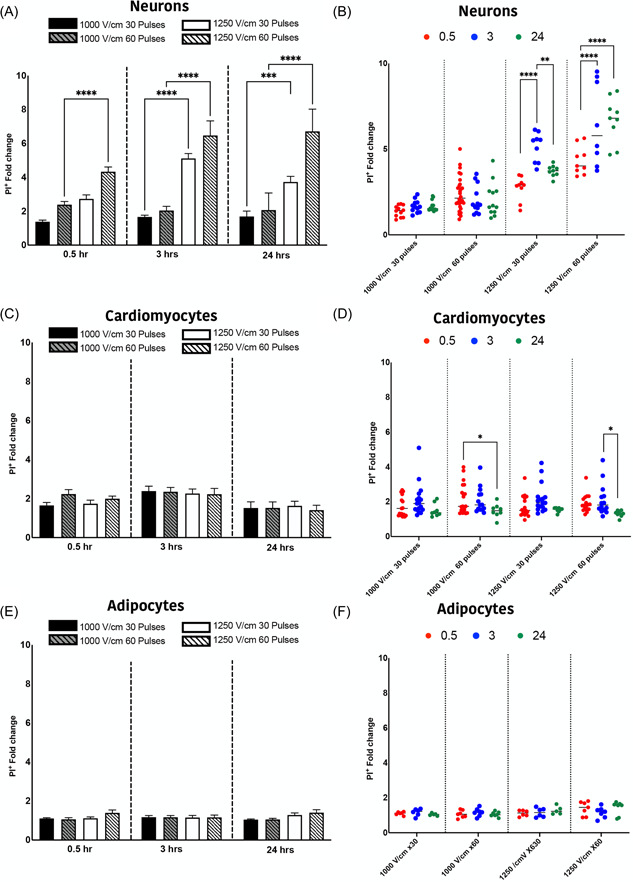
Temporal dynamics of cell death in neurons and cardiomyocytes and adipocytes. The permeability of cells to PI (PI^+^) was measured at 1000 V/cm 30 pulses (in blue), 1000 V/cm 60 pulses (dashed blue), 1250 V/cm 30 pulses (in red) and 1250 60 pulses (dashed red) in neurons (A), cardiomyocytes (C) and cardiac adipocytes (E). The effect of time on PI uptake was examined at 0.5 (red), 3 (blue) and 24 (green) hours for neurons (B), cardiomyocytes (D) and cardiac adipocytes (F). All data shown as mean ± *SEM*. Statistical significance performed using two‐way ANOVA (*p < .05, **p < .005, ***p < .001, ****p < .0001). ANOVA, analysis of variance

Cells in 2D culture were confirmed as positive for the appropriate markers (Figure 2) and were treated with IRE protocols previously established as lethal field thresholds (1000 and 1250 V/cm, 30 or 60),^13^ Neurons exposed to 1250 V/cm showed a significant increase in PI uptake (fold change increase in PI^+^ cells) compared to 1000 V/cm (Figure 3A). The percentage of PI^+^ neurons remained stable across all time points for 1000 V/cm (Figure 3B). However, at 1250 V/cm, there is an increasing cell death over time, particularly at 3 and 24 h (Figure 3B). In contrast, the percentage of PI^+^ cardiomyocytes remained unchanged and independent of applied field strength (Figure 3C). While the percentage of cell death show no significant difference after 3 h, however, it subsequently reduces to below immediate postelectroporation levels at 24 h (Figure 3D). There was significant cell death in adipocytes that was independent of both field strength (Figure 3E) and time (Figure 3F). Comparison of cell sensitivities is summarized in Tables S1 and S2, where neurons and cardiomyocytes show no significant difference at 1000 V/cm (Table S1). Adipocytes at 1000 V/cm at 24 h were significantly different to neurons only (Table S1). At 1250 V/cm, neurons showed a greater PI^+^ cells at both 30 and 60 pulses compared to adipocytes, but only at 60 pulses compared to cardiomyocytes (Table S2). These data demonstrate that neurons have higher susceptibility to cell death in comparison to both cardiomyocytes and cardiac adipocytes at the higher electric field strength of 1250 V/cm.

### 3.2 Neurons are more susceptible to cell damage in comparison to cardiomyocytes

**Figure 4:**
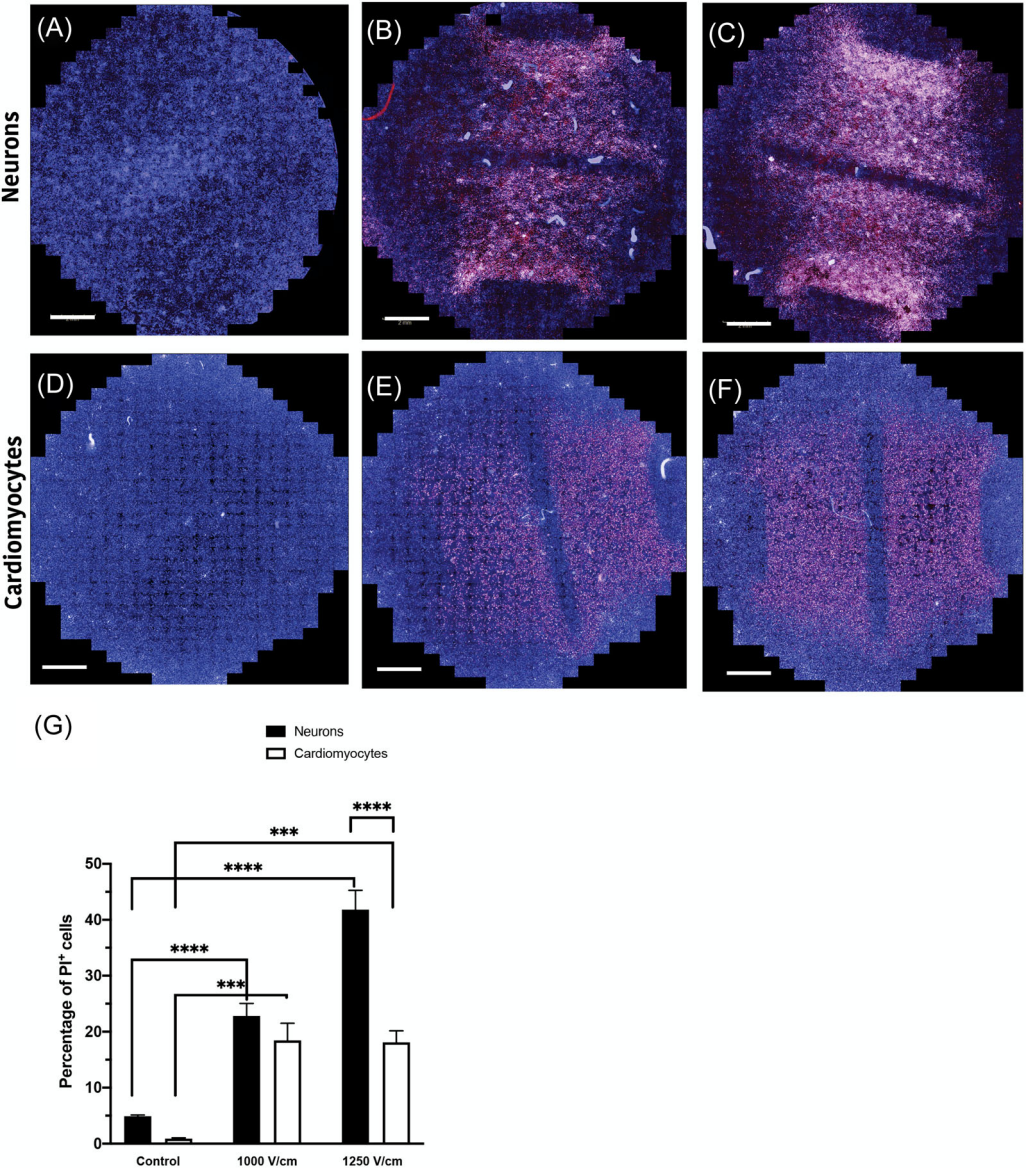
Neurons exhibit increased PI permeability compared to cardiomyocytes. (A) Representative images of control neurons (A) and ablated neurons at 1000 V/cm (B) and 1250 V/cm (C) field strengths, stained with PI. PI permeability were similarly assessed in cardiomyocytes (D) with same treatment (E) 1000 V/cm and 1250 V/cm (F). (G) Neurons and cardiomyocytes show a similar degree of cell death at 1000 V/cm; however, neurons show increased cell death compared to cardiomyocytes at 1250 V/cm. Nuclei counterstained with DAPI. All data shown as mean ± *SEM*. Scale bar at 2 mm. Statistical significance performed using two‐way ANOVA (***p < .001, ****p < .0001). ANOVA, analysis of variance; PI, propidium iodide

The percentage of PI^+^ cells and cell membrane integrity were quantified at 0.5 h after electroporation, with 60 pulses of 1000 or 1250 V/cm field strength, for both neurons (Figure 4A–C) and cardiomyocytes (Figure 4D–F). In both neurons and cardiomyocytes there is a significant increase in cell death (percent PI^+^ cells) in comparison to controls (Figure 4A and 4D). While there is no significant difference at 1000 V/cm field strength, the percentage of PI^+^ cells are significantly higher in neurons compared to cardiomyocytes at 1250 V/cm (Figure 4G). This suggests that neurons at higher field strength are more susceptible to cell damage with electroporation.

**Figure 5:**
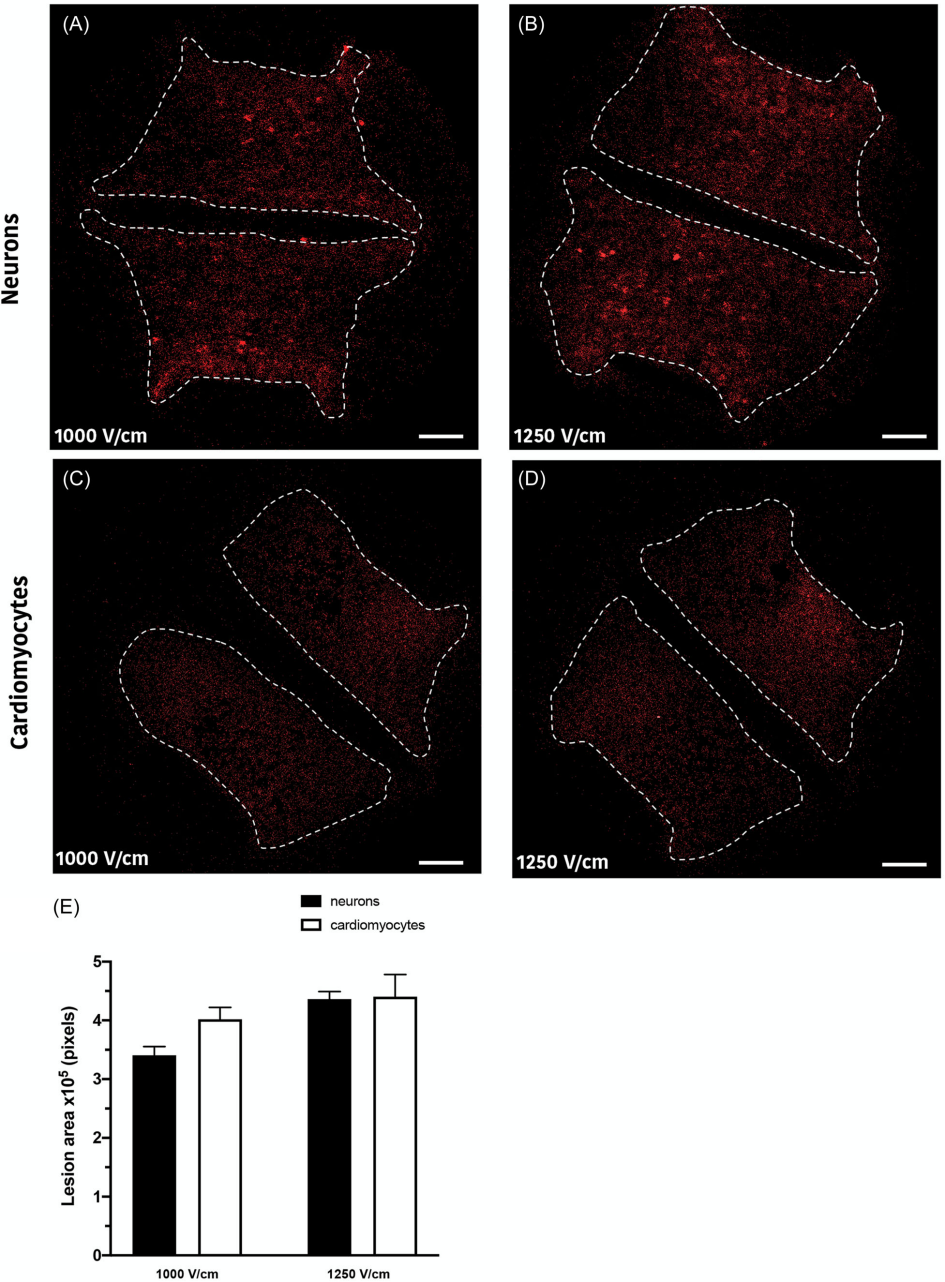
Lesion areas in neurons and cardiomyocytes. (A) Representative images of the lesions are shown for neurons at 1000 V/cm and 1250 V/cm (B) and cardiomyocytes at 1000 V/cm (C) and 1250 V/cm (D) filed strengths. No significant difference was observed between the lesion areas among cell lines (E). Scale bar at 2 mm. All data shown as mean ± *SEM*

The lesion area was determined for neurons (Figure 5A,B) and cardiomyocytes (Figure 5C,D) as a measure of cell death area after electroporation. There was no significant difference in the area ablated across all treatment protocols (Figure 5E). While the percentage of cell death was significantly higher in neurons in comparison to cardiomyocytes, the spread of cell death across the field was found to be similar.

### 3.3 Effect of electroporation on caspase 3/7 activity

**Figure 6:**
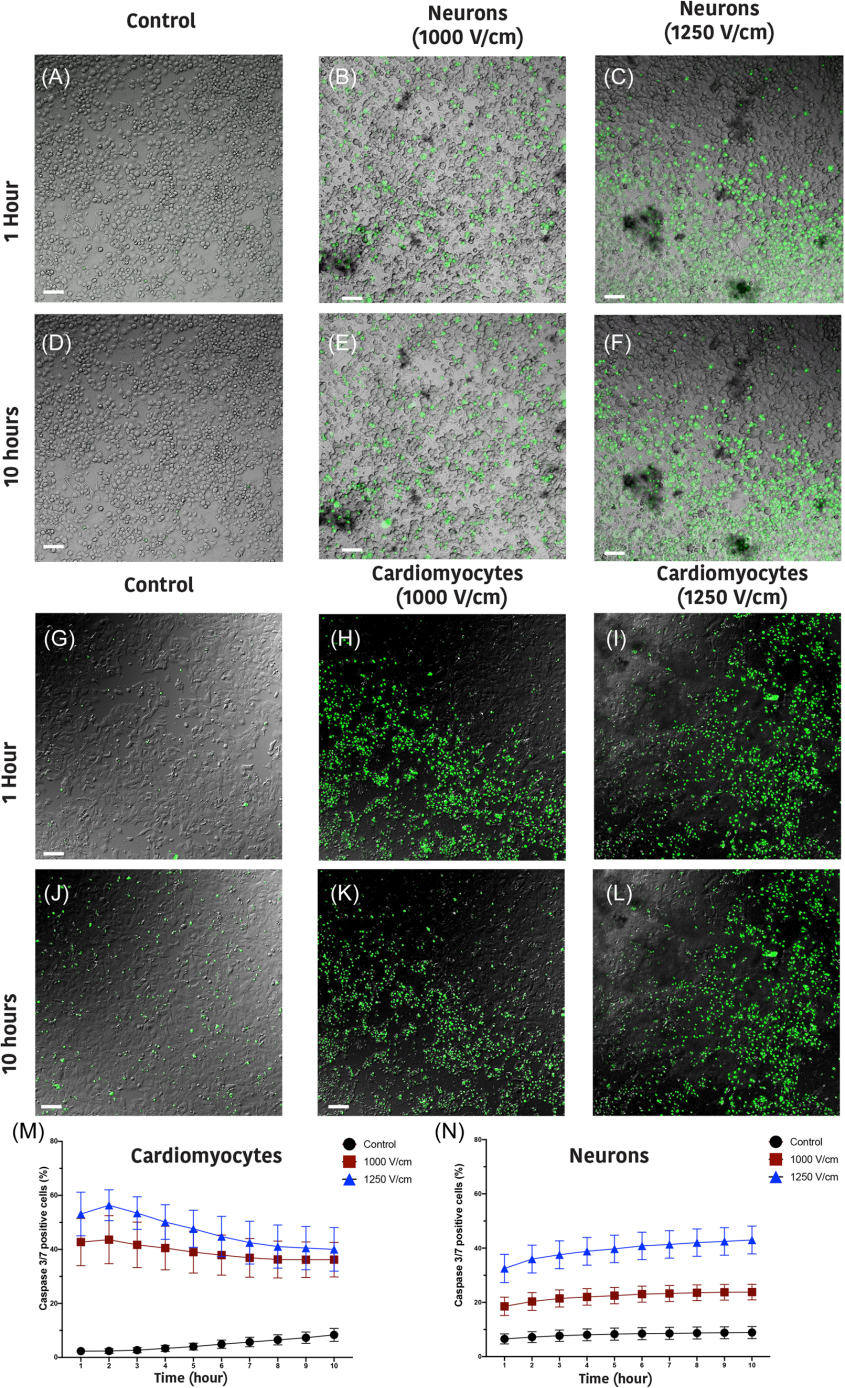
Neurons and cardiomyocytes exhibit caspase activity following electroporation. Representative images of caspase 3/7 activity (in green) in control (A), 1000 V/cm (B) and 1250 V/cm (C) in neurons and in (G), (H), (I), respectively, for cardiomyocytes after 1 h and in (D)–(F) (neurons) and (J)–(L) (cardiomyocytes) after 10 h of electroporation. The percentage of caspase 3/7 positive cells is significantly higher in 1250 V/cm in comparison to 1000 V/cm in both cardiomyocytes (M) and neurons (N) with constant evolution over time. However, Cardiomyocytes showed significantly higher percentage of caspase activity in comparison to neurons. All data shown as mean ± *SEM*

In addition of necrotic cell death, which was observed in the presence of PI, pycnotic type morphology was additionally observed in both neurons and cardiomyocytes, a clear indication of apoptosis. Caspase 3/7 activity was assessed by live‐cell imaging over 10 h postelectroporation. Increased caspase 3/7 activity shows that apoptosis is present as a mechanism of cell death, in addition to necrosis, following electroporation. Caspase activity increased in response to all IRE protocols in neurons (Figure 6B,C) and cardiomyocytes (Figure 6H,I) within 1 h of electroporation compared to control (Figure 6A and 6G). There is a significant interaction effect for caspase 3/7 when comparing treatment (*p* < .001) and cell type (*p* < .001), however, this interaction effect is not significant over time (*p* < .784) (Table S3). Caspase activity remained constant in both neurons (Figure 6E,F) and cardiomyocytes (Figure 6K,L) over the 10‐h time course. Figure 6M shows that there is no significant difference for cardiomyocytes when comparing field strength. However, in neurons, there is a significant difference when comparing field strength (Figure 6N). In addition, these data suggest that the percentage of cell death mediated by caspase activation is significantly higher in cardiomyocytes compared to neurons (Table S3).

### 3.4 Effect of biphasic versus monophasic pulses on cell death in neurons and cardiomyocytes

**Figure 7:**
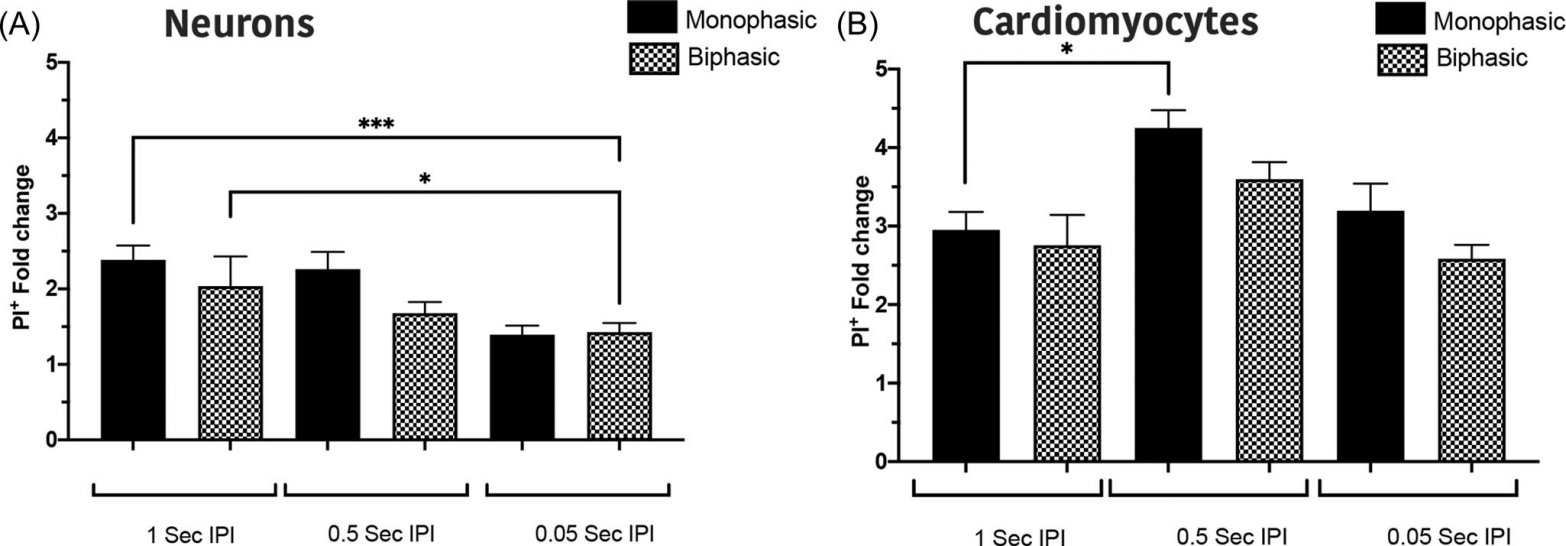
The number of PI^**+**^ cells is similar for biphasic and monophasic pulses. Biphasic pulses of different inter pulse interval were examined in neurons (A) and cardiomyocytes (B). Neurons at 1 s IPI show more PI^+^ cells in comparison to 0.05 IPI with no difference between mono and biphasic pulses. In contrast cardiomyocytes showed increased PI^+^ cells at 0.5 IPI but no significance alteration at 0.05. All data shown as mean ± *SEM*. Statistical significance performed using two‐way ANOVA (*p < .05, ***p < .001). ANOVA, analysis of variance; IPI, inter‐pulse interval; PI, propidium iodide

The effect of different inter‐pulse intervals (IPI) (1, 0.5, and 0.05 s) was examined in addition to comparing biphasic and monophasic pulses. There was no significant difference between cell types (Figure 7A,B) at 1000 V/cm, when comparing biphasic or monophasic pulses regardless of the IPI interval. However, for neurons there is significantly higher percentage of PI uptake for one second, compared to 0.05 s intervals for both waveforms (Figure 7A). The number PI^+^ cardiomyocytes remained stable with the exception of 0.5 s intervals in monophasic pulses (Figure 7B). These results suggest that there is no significant difference between monophasic or biphasic pulses, with neurons being more susceptible to cell death at longer IPI compared to cardiomyocytes.

## 4 DISCUSSION

Our data show that neurons are significantly more susceptible to damage from electroporation than cardiomyocytes, particularly at higher field strengths (>1000 V/cm, Figure 3). Human cardiac adipocytes showed significantly lower susceptibility to damage compared to neurons. In addition, at 1250 V/cm, neurons showed a delayed cell death, with a significant increase in cell death 24 h postelectroporation. In contrast, cardiomyocytes at both 1000 and 1250 V/cm, there was a significant reduction in PI^+^ cells after 24 h indicating the capacity for membrane recovery in cardiomyocytes (Figure 3). This highlights the potential tuneability of ablation via the number of pulses and field strength, as these parameters influence the immediate, delayed, or apparent recovery mechanisms. We also demonstrated that shorter inter pulse intervals (<0.05 s) result in lower cell death, but only in neurons (Figure 7). Furthermore, we illustrate that the 2D lesion sizes were similar for both neurons and cardiomyocytes immediately after treatment. Thus, while IRE is causing more cell death at higher field strengths in neurons compared to cardiomyocytes, the geographical spread of cell death is not significantly different. Monophasic and biphasic waveforms only differed in the effect of inter‐pulse intervals (Figure 7). Importantly, based on the alterations in morphology of both neurons and cardiomyocytes after electroporation, we also determined that the cell death mechanism in neurons and cardiomyocytes occurs both from necrotic (PI^+^) and apoptotic (caspase 3/7 activity) pathways (Figure 6).

Single pulse monophasic IRE ablation has been successfully used for PVI but assessment of long‐term outcomes remains outstanding.^14^ The application of biphasic IRE waveforms for PVI have been successfully demonstrated in the absence of any major adverse effects on the phrenic nerve or oesophagus in both persistent and paroxysmal AF patients.^5^, ^15^, ^16^ While both monophasic and biphasic seem to be effective, the advantages or disadvantages of either waveform on lesion formation is unclear from clinical studies to date and further evaluation is needed. Our data suggest that there is no significant difference in percentage of cell death between monophasic or biphasic for both neurons and cardiomyocytes. But the extent of cell death needs to be further investigated and compared in a more sophisticated 3D model systems.

Tissue selectivity, the dynamics and mechanism of cell death following electroporation in different tissue types, close to an in vivo scenario, is one of the fundamental unanswered questions in this field of study. Recently a study by Hunter et al.^17^ using a similar 2D culture system to that employed here, showed higher susceptibility of ventricular cardiomyocyte to cell damage in comparison to cortical neurons. However, our results indicated that sympathetic like differentiated PC12 neurons (representing neurons similar to the autonomic nervous system) are more susceptible to cell damage compared to atrial cardiomyocytes. The difference observed between studies may reflect the cell types used in each case.

Based on our data, at higher field strength, the percentage of cell damage is greater in neurons compared to cardiomyocytes but with a similar lesion size for both. This finding can be extremely important as these results imply that at higher field strength, cardiomyocytes are more resilient and undergo some degree of reversible electroporation. In our study, we show that while a fraction of cardiac cells die almost immediately after the treatment, regardless of the electric field, the membrane integrity of a substantial number of cells significantly recovers by 24 h, reflecting that an electric field higher than 1250 V/cm is required to diminish this reversibility capacity. This reversibility was also observed in other experiments in the early time points after application of conventional IRE pulses.^18^, ^19^ However, in these studies the cells (cancer) eventually lost their membrane integrity after 8 h and died. Our results showed that in contrast to reversibility observed in cardiomyocytes, neurons showed similar cell damage at immediate and 24 h time points with no reversibility. This highlighted the importance of choosing appropriate observation times to assess cell ablation following electroporation. Previous studies have pointed out the significance of observational times for histological evaluations of the ablated tissues treated with conventional IRE.^20^ Therefore, in clinical applications the optimal monitoring times after the application of electroporation is essential for accurate measurement of cell death.

Although the definitive goal of ablation is to cause selected cell damage in a specified volume, it is essential to understand the mechanism of cell death caused by the treatment. Cell death pathways are dependent to multiple variables such as field strength, pulse number and cell type.^21^ The type of cell death depends on the activation of specific pathways that can ultimately trigger either inflammatory or immunogenic responses.^22^ We observed that a proportion of cells exhibits caspase 3/7 activity demonstrating that apoptotic pathways are also activated. In line with the significant increase in PI^+^ cells at higher field strength (1250 V/cm) in neurons, a higher proportion of dead cells also undergo apoptosis in comparison to 1000 V/cm. This suggest that in neurons higher electric field strength can push cell death towards apoptosis. The level of caspase activity in both cell types remain relatively constant over a 10‐h time course. We show that the percentage of caspase 3/7 activity is significantly higher in cardiomyocytes than in neurons for both fields tested. This may demonstrate that a higher proportion of cell death occurs via apoptosis in cardiomyocytes, however neurons at higher electric fields also show an increase trend in caspase activity, reaching a similar level over time. It has been proposed that apoptosis or silent cell death triggers lower inflammatory or immunogenic responses which makes it a more desirable cell death modality, which has been noted in other studies also following IRE.^19^, ^23^ While apoptosis can also trigger immunogenic responses under certain conditions, it should also be noted that nonlethal caspase activation is reported in mature muscle cells and primary myoblast cell lines.^24^, ^25^ Thus, further studies are required for fully elucidate the effect of conventional IRE on cell death pathways.

## 5 LIMITATIONS

In this study, an adherent monolayer of neuronal and cardiac‐related cell types facilitated the initial development of optimized parameters allowing the iterative design of pulse protocols. However, while these cells are a very close representative cell line for neurons and cardiac muscle residing in GPs and adjacent tissues, they are limited by the lack of whole cardiac tissue 3D geometry. Another limitation of the current approach is that the conductivity of blood is significantly lower than a typical tissue culture medium which we expect would affect the electric field thresholds. We have no reason to believe that the relative differences observed with IRE in cell culture would not be maintained in vivo despite the alternated conductivity. However, further data is required to fully answer this important question. The results from this study add new knowledge to this area of research and highlight the potential for cell selectivity with IRE. Future studies with three‐dimensional cell culture constructs and more in‐vivo/ex vivo studies are required to extend the parameters identified from our current model. In addition, with more complex geometric models the effect of IRE on axonal processes versus cell bodies could be studied further.

## 6 CONCLUSIONS

Our results highlight the potential tissue selectively and the cell death pathway selectivity of IRE, depending on the field strength, pulse number and inter‐pulse interval. We have clearly shown that at higher field strengths, neurons are more susceptible to cell death compared to both cardiomyocytes and adipocytes. Immediate, delayed cell death and apparent partial reversibility in both neurons and cardiomyocytes at appropriate time of assessment have potential implications for the timing of clinical assessment after delivery of electroporation therapy. We demonstrate that neurons at high field strength have delayed cell death, while cardiomyocytes showed significant reversibility of electroporation effects 24 h after treatment. The monophasic and biphasic waveform of the electroporation showed no significant effect in both neurons and cardiomyocytes. Investigation of the cell death mechanism after electroporation, showed that while sudden cell death/necrosis occurs in both neurons and cardiomyocytes, the level of caspase 3/7 enzyme in neurons is significantly higher, suggesting a possible increase in apoptotic cell death compared to cardiomyocytes. These results provide new insight in the field of IRE but must be explored further to extrapolate to clinical outcomes.

## CONFLICTS OF INTEREST

The authors declare no conflicts of interest.

## Supporting information

Supplementary information.

## Data Availability

The data underlying this article will be shared on reasonable request to the corresponding author.
