# Supplementary material for: Establishing electroporation thresholds for targeted cell specific cardiac ablation in a 2D culture model
Source: J Cardiovasc Electrophysiol. 2022 Aug 16;33(9):2050–61. doi: 10.1111/jce.15641 (PMC9543844; doi:10.1111/jce.15641)
Supplement: Supplementary file 1 — Supplementary information. [file JCE-33-2050-s001.docx]

**Supplemental Table 1.** Comparison of cell death (PI^+^ fold change) between neurons, cardiomyocytes, and cardiac adipocytes at 0.5-, 3- and 24-hour time point at 1000 V/cm field strength.

| **1000 V/cm (pulses, hr)** | **Mean ±SEM**  **1^st^** | **Mean ±SEM**  **2^nd^** | **P value** |
| --- | --- | --- | --- |
| HL-1 vs PC12 (30, 0.5) | 1.65±0.15 | 1.38±0.09 | 0.6165 (ns) |
| Hl-1 vs Adipocyte (30, 0.5) | 1.65±0.15 | 1.12±0.04 | 0.2808 (ns) |
| PC12 vs Adipocyte (30, 0.5) | 1.38±0.09 | 1.12±0.04 | 0.7334 (ns) |
|  |  |  |  |
| HL-1 vs PC12 (30, 3) | 2.39±0.25 | 1.66±0.10 | 0.0352 |
| Hl-1 vs Adipocyte (30, 3) | 2.39±0.25 | 1.17±0.08 | 0.025 |
| PC12 vs Adipocyte (30, 3) | 1.66±0.10 | 1.17±0.08 | 0.3822 (ns) |
|  |  |  |  |
| HL-1 vs PC12 (30, 24) | 1.52±0.08 | 1.69±0.9 | 0.8337 (ns) |
| Hl-1 vs Adipocyte (30, 24) | 1.52±0.08 | 1.05±0.03 | 0.4301 (ns) |
| PC12 vs Adipocyte (30, 24) | 1.69±0.9 | 1.05±0.03 | 0.2312 (ns) |
|  |  |  |  |
| HL-1 vs PC12 (60, 0.5) | 2.88±0.24 | 2.39±0.19 | 0.0430 |
| Hl-1 vs Adipocyte (60, 0.5) | 2.88±0.24 | 0.01±0.08 | <0.001 |
| PC12 vs Adipocyte (60, 0.5) | 2.39±0.19 | 0.01±0.08 | 0.0001 |
|  |  |  |  |
| HL-1 vs PC12 (60, 3) | 2.35±0.22 | 2.04±0.24 | 0.5722 (ns) |
| Hl-1 vs Adipocyte (60, 3) | 2.35±0.22 | 1.12±0.09 | 0.0030 |
| PC12 vs Adipocyte (60, 3) | 2.04±0.24 | 1.12±0.09 | 0.0357 |
|  |  |  |  |
| HL-1 vs PC12 (60, 24) | 1.52±0.08 | 2.07±0.29 | 0.1456 (ns) |
| Hl-1 vs Adipocyte (60, 24) | 1.52±0.08 | 1.06±0.05 | 0.3639 (ns) |
| PC12 vs Adipocyte (60, 24) | 2.07±0.29 | 1.06±0.05 | 0.0121 |

Statistical significance performed using two-way Anova. ns stand for not significant. 1^st^ reflet to the mean±SEM of the first cell line mentioned on the raw and 2^nd^ reflect the mean±SEM of the second cell line mention on the raw.

| **1250 V/cm (pulses, hr)** | **Mean ±SEM**  **1^st^**  **1st** | **Mean ±SEM**  **2^nd^** | **P value** |
| --- | --- | --- | --- |
| HL-1 vs PC12 (30, 0.5) | 1.71±0.23 | 2.72±0.24 | 0.0154 |
| Hl-1 vs Adipocyte (30, 0.5) | 1.71±0.23 | 1.12±0.07 | 0.2679 (ns) |
| PC12 vs Adipocyte (30, 0.5) | 2.72±0.24 | 1.12±0.07 | 0.0008 |
|  |  |  |  |
| HL-1 vs PC12 (30, 3) | 2.26±0.23 | 5.12±0.28 | <0.0001 |
| Hl-1 vs Adipocyte (30, 3) | 2.26±0.23 | 1.12±0.11 | 0.0173 |
| PC12 vs Adipocyte (30, 3) | 5.12±0.28 | 1.12±0.11 | <0.0001 |
|  |  |  |  |
| HL-1 vs PC12 (30, 24) | 1.63±0.06 | 3.72±0.11 | <0.0001 |
| Hl-1 vs Adipocyte (30, 24) | 1.63±0.06 | 1.23±0.11 | 0.7013 (ns) |
| PC12 vs Adipocyte (30, 24) | 3.72±0.11 | 1.23±0.11 | <0.0001 |
|  |  |  |  |
| HL-1 vs PC12 (60, 0.5) | 2.00±0.13 | 4.33±0.27 | <0.0001 |
| Hl-1 vs Adipocyte (60, 0.5) | 2.00±0.13 | 1.34±0.15 | 0.2512 (ns) |
| PC12 vs Adipocyte (60, 0.5) | 4.33±0.27 | 1.34±0.15 | <0.0001 |
|  |  |  |  |
| HL-1 vs PC12 (60, 3) | 2.23±0.29 | 6.47±0.86 | <0.0001 |
| Hl-1 vs Adipocyte (60, 3) | 2.23±0.29 | 1.12±0.12 | 0.0216 |
| PC12 vs Adipocyte (60, 3) | 6.47±0.86 | 1.12±0.12 | <0.0001 |
|  |  |  |  |
| HL-1 vs PC12 (60, 24) | 1.41±0.06 | 6.71±0.44 | <0.0001 |
| Hl-1 vs Adipocyte (60, 24) | 1.41±0.06 | 1.40±0.15 | 0.9997 (ns) |
| PC12 vs Adipocyte (60, 24) | 6.71±0.44 | 1.40±0.15 | <0.0001 |

**Supplemental Table 2.** Comparison of cell death (PI^+^ fold change) between neurons, cardiomyocytes and cardiac adipocytes at 0.5, 3 and 24 hour time point at 1250 V/cm field strength.

Statistical significance performed using two-way Anova. ns stand for not significant. 1^st^ reflet to the mean±SEM of the first cell line mentioned on the raw and 2^nd^ reflect the mean±SEM of the second line mention on the raw.

**Supplemental Table 3.** Repeated measure Anova analysis of caspase 3/7 activity.

|  | **df** | **Sum Sq** | **Mean Sq** | **F-value** | **Pr (>F)** |
| --- | --- | --- | --- | --- | --- |
| **Cell type** | 1 | 3753 | 3753 | 69.134 | <0.001 |
| **Treatment** | 2 | 48855 | 24428 | 450.029 | <0.001 |
| **Time** | 9 | 30 | 3 | 0.061 | ~1.000 |
| **Cell type: Treatment** | 2 | 2658 | 1329 | 24.482 | <0.001 |
| **Cell type: Time** | 9 | 642 | 71 | 1.314 | 0.237 |
| **Treatment: Time** | 18 | 200 | 11 | 0.205 | ~1.000 |
| **Cell type: Treatment: Time** | 18 | 704 | 39 | 0.721 | 0.784 |
| **Residuals** | 118 | 6405 | 54 |  |  |
